# The tissue microarray OWL schema: An open-source tool for sharing tissue microarray data

**DOI:** 10.4103/2153-3539.65347

**Published:** 2010-07-13

**Authors:** Hyunseok P. Kang, Charles D. Borromeo, Jules J. Berman, Michael J. Becich

**Affiliations:** 1 Department of Pathology, Roswell Park Cancer Institute, Elm and Carlton St, Buffalo 14263, NY, USA,; 2 Department of Biomedical Informatics, University of Pittsburgh, 5150 Centre Avenue, Pittsburgh, PA 15232, USA; 3 No Affliations

**Keywords:** Ontology, OWL, tissue microarray

## Abstract

**Background:**

Tissue microarrays (TMAs) are enormously useful tools for translational research, but incompatibilities in database systems between various researchers and institutions prevent the efficient sharing of data that could help realize their full potential. Resource Description Framework (RDF) provides a flexible method to represent knowledge in triples, which take the form Subject-Predicate-Object. All data resources are described using Uniform Resource Identifiers (URIs), which are global in scope. We present an OWL (Web Ontology Language) schema that expands upon the TMA data exchange specification to address this issue and assist in data sharing and integration.

**Methods:**

A minimal OWL schema was designed containing only concepts specific to TMA experiments. More general data elements were incorporated from predefined ontologies such as the NCI thesaurus. URIs were assigned using the Linked Data format.

**Results:**

We present examples of files utilizing the schema and conversion of XML data (similar to the TMA DES) to OWL.

**Conclusion:**

By utilizing predefined ontologies and global unique identifiers, this OWL schema provides a solution to the limitations of XML, which represents concepts defined in a localized setting. This will help increase the utilization of tissue resources, facilitating collaborative translational research efforts.

## INTRODUCTION

Tissue microarrays (TMAs) are collections of hundreds of tissue cores arrayed into a single paraffin histology block, typically containing between 100 and 1000 core tissue samples. Each TMA block can be sectioned and mounted onto glass slides, producing hundreds of nearly identical slides. By allowing researchers to simultaneously measure a marker in hundreds of specimens arrayed on a single slide, they conserve an enormous amount of time, money, and reagents.[1] Most importantly, they amplify and extend the use of limited tissue resources which are irreplaceable, enabling high-throughput controlled studies on large cohorts of tissues. Another advantage of TMA experiments is that specimens from different donor tissue blocks are treated to identical incubation times, temperatures, and washing conditions, standardizing the experiment and making it much easier to compare markers between the different core sections.

As more and more studies are performed using high-throughput technologies such as DNA microarrays, TMA technology has also proven to be a valuable tool for high-throughput validation of marker genes identified in these experiments.[2,3]

Because a single paraffin TMA block can be sectioned into nearly identical glass slides and dispensed to many different laboratories, this technology also assists in collaboration and sharing of resources. However, this is also accompanied by increased complexity; different laboratories may use different experimental protocols and instruments and capture data using different data elements, formats, and structures. There have been some efforts to address this by developing Common Data Elements (CDE) for collaborative tissue resources.[4,5] Although integrating the TMA data from different laboratories would dramatically increase the value of experimental results and reduce redundant testing, it is difficult to put this into practice due to incompatibilities in datasets between laboratories using the different database or information systems that are available.[6] Compounding this is the fact that most of today's Laboratory Information Systems (LISs) are not prepared for this type of data.[7] Together, these factors prevent the true scientific value of this technology from being realized. Expanding upon the TMA data exchange specification previously described by Berman *et al*,[8] we have designed an OWL (Web Ontology Language) schema that will help researchers share experimental and clinical data on TMA experiments.

## METHODS

Before discussing the features of OWL, an introduction to Resource Description Framework (RDF) is necessary. OWL is built on top of RDF concepts. As such, an understanding of RDF is essential for describing OWL. RDF provides a flexible method to represent knowledge by deconstructing it into small pieces called triples. Triples, also known as statements, take the form Subject-Predicate-Object and can be regarded as being similar to simple sentences. For example, the statement “tissue core B15 is derived from block RP2007-189” can be broken down into (tissue core B15) (Subject) (is derived from) (Predicate) (block RP2009-189) (Object). In RDF, the subjects, predicates, and objects are names for resources which represent some entity, such as a person, website, book, tissue block, etc. These names are usually Uniform Resource Identifiers (URIs) and are global in scope, meaning that they always refer to the same entity. The most well-known format for URIs is the URL, although it can be anything that the creators of files agree upon, such as an International Standard Book Number (ISBN). Objects can also be literals such as numbers or text strings, for example (block RP2009-258) (Subject) (number of cores) (Predicate) (100) (Object). Subjects and objects can be instances of RDF classes, while predicates are RDF properties. RDF classes, properties, and the relationships between them are defined in RDF schema documents.

OWL extends the expressivity of the RDF schema by describing more complicated relationships. OWL allows a schema designer to connect two concepts in an inverse relationship. For example, the predicate “has child” in the simple relationship (father Dad1) (Subject) (has child) (Predicate) (child Child1) has a natural inverse relationship “has father”. The inverse relationship is expressed by reversing the subject and object: (child Child1) (has father) (father Dad1). Although RDF allows one to describe both relationships separately, it is OWL that allows the two to be described as inverses of one another. OWL also allows for automated processing of its data since it is more semantically stringent than XML or RDF.

The goal of this effort was to design a minimal OWL schema that defined classes and properties specific to describing TMAs and experiments performed on them, adhering to the spirit of the guiding principles set forth by Berman *et al*.[8] This reduces the number of data elements described and simplifies the document. In many cases, more general concepts are described in other ontologies, which users can utilize. For example, instead of defining classes such as “#pathology_report” or “#organism” in our schema, we suggest that users generate instances of classes defined in external ontologies, such as the NCI Thesaurus (http://ncicb.nci.nih.gov/xml/owl/EVS/Thesaurus.owl) which includes these classes. In addition to simplifying the data specification, this provides a mechanism to integrate data from these OWL documents and other documents utilizing those ontologies.

TMAs represent data obtained from a wide variety of diseases and experimental conditions. However, there are several central concepts unifying the artifacts generated as part of the TMA manufacturing process. For the purposes of this exercise, we focused on the central issue of relating TMA concepts (i.e. a core to a block, a core to a slide, etc.) to one another in a semantically significant manner. We avoided attempting to describe concepts in huge topics such as assays, diagnosis, and anatomy, many of which currently have their own large-scale vocabulary or ontology projects.[9] Although TMAs are ultimately used for disease research and diagnosis, these topics are far too broad to include in a singular OWL scheme, in addition to not being limited to TMAs. Therefore, we kept the scope of the schema focused on concepts central to TMAs. We also wanted to present a focused and pragmatic description that others can follow to create their own instances of OWL files from their data. This is not intended to be an exhaustive exploration.

There are several methods available for extracting TMA data from data sources (i.e. database, files, etc.). In general, the extraction approach should follow these steps:

Collect the data to be represented in the OWL file (e.g. SQL query);

Store the collected data in a well-defined structure (e.g. an XML file);

Convert the well-defined structure into a set of OWL files (e.g. an XSLT transformation).

The actual methods employed to implement these steps will depend largely on the starting data source format. The easiest approach involves collecting the data in a well-defined XML file. Next, convert the XML file to an OWL file using XSLT. An XSLT transformation provides the best method for converting data into OWL. Whereas the conversion process is fairly straightforward, the process of creating identifiers for the objects within the TMA OWL file is more involved.

TMAs have a long life and are reused thousands of times. In addition, the samples from a TMA are typically dispersed to several institutions/laboratories for various experiments. The dispersion of samples may occur over the course of several years. Therefore, it is essential to have a centralized identifier to describe the TMA. Unfortunately, these centralized identifiers are a source of intense debate within the RDF community. For openness and ease of use, we have elected to use the Linked Data[10] format, which grew from the Linking Open Data (LOD) project. The LOD attempts to link data from a wide variety of data sources including entertainment, social networking, and life sciences. To use the Linked Data format, one must define a set of URIs for the data. One must also ensure that the URIs are resolvable and supply useful information about the data represented by the URI. The Linked Data format does not impose any specific format, but does supply some suggestions that are given below:

Define URIs in an HTTP namespace under one's control. Do not define them in someone else's namespace.

Keep URIs stable and persistent. Changing URIs will break any already established links, so it is advisable to devote some extra thought to them at an early stage.

In general, one needs to use a primary key inside a URI to make sure that each URI is unique. Whenever possible, use a key that is meaningful inside your domain. For example, when dealing with books, making the ISBN number part of the URI is better than using the primary key of an internal database table. If one is representing a TMA ordered from National Mesothelioma Virtual Bank (NMVB), use the NMVB identifier in the URI.

For example, assume one's domain is *www.institutionXYZ.org* and within the organization there is an RDF data file containing a list of TMAs that can be found at http://www.institutionXYZ.org/tma/rdf. The URI of a TMA with identifier 123456 would then be:

http://www.institutionXYZ.org/tma/rdf/123456

## RESULTS

The OWL schema document can be found at *http://bioportal.bioontology.org/ontologies/42764* and is also provided as a separate file (tma_minimal.owl) with this article. The classes and properties defined in this document are listed in Table 1. As shown, there are paired properties describing the bidirectional relationship between tma, block, slide, and core. The "top-down" properties (includes_block, block_includes_core) should be used in OWL documents that include the entire hierarchical structure of a TMA, while the "bottom-up" properties (included_in_tma, cut_from_block) can be used when a resource refers to a parent block or TMA instance in a different document. repository_product and experimental_component are abstract parent classes which are necessary to ensure that the various classes and properties are used according to OWL syntax rules.

**Table 1:** RDF classes and properties

Resource type	Resource
Class	repository_product
	experimental_componen
	tma
	block
	slide
	core_in_block
	core_on_slide
Object property	includes_block
	included_in_tma
	block_includes_core
	included_in_block
	slide_includes_core
	affixed_to_slide
	includes_slide	
	cut_from_block	
	derived_from_core
	donor_block
Datatype property	status
	level
	size
	thickness
	core_spacing
	location
	diagnosis
	clinical_annotation
	drill_site
	protocol
	report_link
	control
	result
	repository
	assay

**Figure 1:**
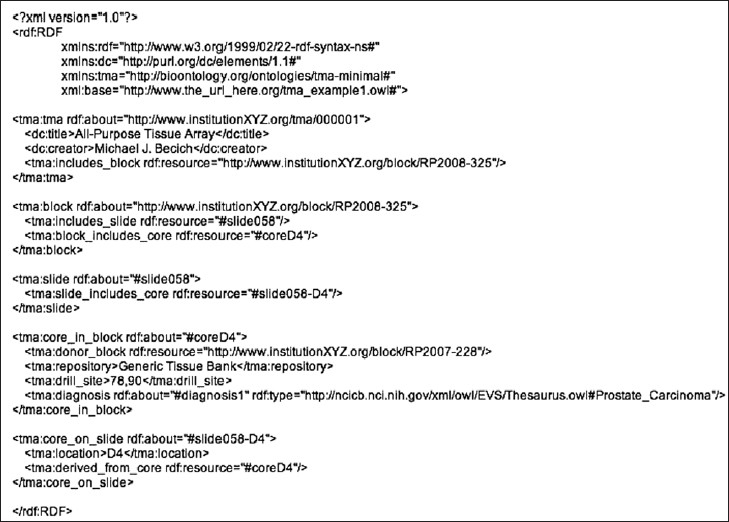
Example OWL file; this example uses the TMA OWL schema to describe the basic elements of a simplified TMA

Figure 1 shows an example of a valid OWL file utilizing this schema. In this example, the first and second lines specify that the document is XML, and that it is an RDF resource, respectively. Lines 3, 4, and 5 assign shorthand prefixes for the namespace documents that will be referred to. When one of these prefixes is followed by a colon, it can be expanded into the full URI of the namespace document. Line 6 specifies the base URI of this document. Resource identifiers that are simply preceded by a “#” are assumed to belong to this base URI and expanded accordingly. Sample expansions are listed in Table 2.

**Table 2:** Sample expansions

Shorthand	Expanded
#slide058	http://www.the_url_here.org/tma_example1.rdf#slide058
tma:block	http://bioontology.org/ontologies/tma-minimal#block
dc:title	http://purl.org/dc/elements/1.1#title

There are some shorthand conventions in RDF/XML that one needs to be aware of to read it correctly. Each block of text encapsulated by opening and closing Class names ('<tma:tma.... </tma:tma>' – these are separated by lines in this case for readability) creates an instance (the URI of which is declared in “rdf:about”) of that class. The opening and closing tags also encompass a group of predicate-object pairs that share the same subject (the resource following “rdf:about”). Hence, the first block represents the first four triples listed in Table 3. The first triple states that “type” of the resource “tma/000001” is the “tma” class (creating an instance of the class in the process), the second and third describe the “title” and “creator”, and the fourth states that it includes the resource “http://www.institutionXYZ.org/block/RP2008-325”. In this case, the TMA and block URIs simulate the existence of data files at the respective addresses, while those for slide, core_in_block, and core_on_slide are within the example file itself. The “tma:diagnosis” line illustrates how to use the NCI ontology to assign a diagnosis to a resource. The clinical_annotation property would be utilized in a similar fashion, most likely by pointing to a separate resource that includes these data, or generating a blank node that would have the necessary property/value pairs. The latter option requires a separate schema for the clinical data elements, which might initially be project specific but could be updated and merged with other schema as the field matures.

**Table 3:** List of triples

Subject	Predicate	Object
tma/000001	owl:Class	tma:tma
tma/000001	dc:title	“All-Purpose Tissue Array”
tma/000001	dc:creator	Michael J. Becich
tma/000001	tma:includes_block	block/RP2008-325
block/RP2008-325	owl:Class	tma:block
block/RP2008-325	tma:includes_slide	#slide058
block/RP2008-325	tma:block_includes_core	#coreD4
#slide058	owl:Class	tma:slide
#slide058	tma:slide_includes_core	#slide058-D4
#coreD4	owl:Class	tma:core_in_block
#coreD4	tma:donor_block	block/RP2007-228
#coreD4	tma:repository	“Generic Tissue Bank”
#coreD4	tma:drill_site	“78,90”
#coreD4	tma:diagnosis	#diagnosis1
#diagnosis1	rdf:type	http://ncicb.nci.nih.gov/xml/owl/EVS/Thesaurus.owl#Prostate_Carcinoma
#slide058-D4	owl:Class	tma:core_on_slide
#slide058-D4	tma:location	“D4”
#slide058-D4	tma:derived_from_core	#coreD4

**Figure 2:**
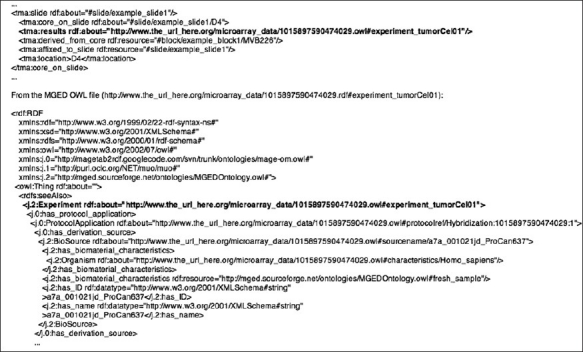
MGED example; this example illustrates one way to connect a file describing a TMA to a data file using a different ontology

In the supplementary data, we have included example files (which can be viewed using any text viewer) for using XSLT transformation (tma_rdf.xsl) to convert data in an XML file (mvbtma1.xml) from the NMVB project[11] to RDF (mvbtma1.rdf). These examples also simulate the existence of RDF files that catalog the various identifiers. These, and the example described in Figure 2, are not exhaustive explorations, but are rather intended to demonstrate some of the ways in which the schema can be utilized.

As mentioned previously, TMAs are a long-term experimental resource. As such, they potentially represent the samples used in several experiments in a wide variety of assays. High-throughput assays like genomic microarrays are commonly employed by researchers to explore a wide range of biological questions and samples included in TMAs are frequently used in these microarray experiments. To further demonstrate the utility of the TMA OWL file, we present an excerpt of the TMA OWL connecting a core on a slide to its microarray experiment results. The results are represented using the MGED Ontology,[12] an OWL ontology used to provide well-defined microarray results. In this case, the MGED Ontology is applied to an existing dataset from the caArray repository (https://cabig.nci.nih.gov/tools/caArray).[13] The MGED OWL data are presented here for illustrative purposes but could be easily derived from the caArray data.

Figure 2 demonstrates how to connect data from an example TMA OWL file to a sample MGED OWL file. In the TMA OWL file, a relationship is established between the core_on_slide (#slide/example_slide1/D4) and the experiment conducted in the MGED OWL file (http://www.the_url_here.org/microarray_data/1015897590474029.owl#experiment_tumorCel01). This same approach could be used to connect the TMA OWL file to an RDF or OWL file containing instances of other types of assay results.

## DISCUSSION

Various efforts have been made to define data exchange standards for TMA data. One obstacle in doing this is the inherent complexity and vast diversity of clinical and histopathologic data that can accompany TMAs. In order to address these issues, Berman *et al*, described an XML TMA data exchange specification that focused on a generic, extensible format that was easy to implement.[8] There have been some implementations of this, in one case building upon the original specification to design a system more closely suited to the needs of a specific collaborative tissue resource.[14–16] In addition, Lee *et al*, designed a more elaborate system, Tissue Microarray-Object Model (TMA-OM). They then implemented it in a web-based database application called Xperanto-TMA,[17] as well as TMA-TAB, a spreadsheet-based data exchange format integrated with the database.[18] This data model, while being comprehensive, is also very complex, which creates a barrier to entry and limits users to the applications that are based on it. While these can be useful as standalone programs and forms, they are relatively inflexible and cannot utilize externally defined standard vocabularies, which also restricts interoperability with other applications based on these standards.

The limitation of XML in the context of data sharing is that although the extensible tags provide a format for representing metadata that humans can understand, there must be some agreement on these tags before they are useful for sharing data. In essence, the requirement for standards still exists; this decision has been left up to the users. OWL, on the other hand, is a format that can draw on preexisting standards and definitions. In general, XML files represent concepts defined by their authors in a localized setting (i.e. an institution, laboratory, a particular application, etc). As a result, the precision of these definitions depends on the amount of time invested by the authors. In many cases, ambiguity in XML arises because XML is not designed to stringently define concepts. XML is primarily intended to exchange information. On the other hand, RDF is designed to reduce ambiguity while defining properties and concepts within a domain. In XML, a tag (or property) is simply a string that denotes a data point: <average_count>3.5</average_count>. In this example, <average_count> is the XML property and “3.5”is the data point. In general, XML will not provide an adequate definition describing the concepts. In this example, average_count represents a mathematical concept with a calculated value of 3.5. Without a clear definition of “average_count”, another person utilizing the XML file would need to know how the “average_count” was calculated. The average_count could represent a simple mean, a weighted average, or a value from an assay's software package. (Another example of this ambiguity exists in the financial world. Most financial papers have price/earnings ratios yet they all have varying methods for calculating the ratio.[19] ) The lab creating the XML file knows precisely what the average_count represents, but the precise definition is lost on a third party.

OWL is designed to reduce ambiguity by providing facilities for defining concepts within files and for defining concepts universally for use in files. In addition, OWL can import vocabularies and concepts from RDF structures. There are several general purpose RDF structures available to provide the basis for new OWL or RDF (RDF/OWL) files:

- Dublin Core Metadata Initiative (dcmi)
- Friend of a Friend (foaf)
- Simple Knowledge Organization System (skos)

These RDF structures provide support for representing bibliographic information, basic labels, information about people, and information about concepts. In addition, there are several organizations creating RDF (or OBO/OWL) structures on a wide array of topics including diseases, taxonomy, phenotypes, etc. These structures are collected by different repositories such as:

- National Center for Biomedical Ontology
- Open Biomedical Ontologies

Centralizing the RDF/OWL structures allows the organizations to define concepts and refine these definitions to reduce ambiguity. These repositories also provide versioning capabilities to support refinements and corrections. By centralizing RDF/OWL structures, an organization can create permanent definitions for concepts. This encourages adoption of the standardized concepts by both existing users and new adopters. Unlike an assay representing a snapshot of data at a particular point in time, TMAs represent a permanent real-life entity. Consequently, a data structure that defines concept definitions in a permanent and universal manner (e.g. RDF/OWL) is preferable to a more transient and localized data structure (e.g. XML). Global unique identifiers and global concept definitions allow true portability, freeing up users to exchange data without prior agreement on data elements, etc. Therefore, with a suitable schema, one institution or investigator may make their data available in an OWL document that other individuals can then utilize. This makes OWL an ideal candidate for designing a framework for the sharing of clinical and test data on TMAs. As described above, such a data sharing framework will create substantial value by greatly facilitating collaborative studies utilizing TMA resources.
